# A Novel Immunoreagent for the Specific and Sensitive Detection of the Explosive Triacetone Triperoxide (TATP)

**DOI:** 10.3390/bios1030093

**Published:** 2011-07-07

**Authors:** Maria Astrid Walter, Ulrich Panne, Michael G. Weller

**Affiliations:** 1 BAM Federal Institute for Materials Research and Testing, Richard-Willstätter-Strasse 11, D-12489 Berlin, Germany; E-Mails: astrid.walter@bam.de (M.A.W.); ulrich.panne@bam.de (U.P.); 2 Chemistry Department, Humboldt-Universität zu Berlin, Brook-Taylor-Strasse 2, D-12489 Berlin, Germany

**Keywords:** organic peroxides, terrorism, biosensor development

## Abstract

Triacetone triperoxide (TATP) is a primary explosive, which was used in various terrorist attacks in the past. For the development of biosensors, immunochemical µ-TAS, electronic noses, immunological test kits, or test strips, the availability of antibodies of high quality is crucial. Recently, we presented the successful immunization of mice, based on the design, synthesis, and conjugation of a novel TATP derivative. Here, the long-term immunization of rabbits is shown, which resulted in antibodies of extreme selectivity and more than 1,000 times better affinity in relation to the antibodies from mice. Detection limits below 10 ng L^−1^ (water) were achieved. The working range covers more than four decades, calculated from a precision profile. The cross-reactivity tests revealed an extraordinary selectivity of the antibodies—not a single compound could be identified as a relevant cross-reactant. The presented immunoreagent might be a major step for the development of highly sensitive and selective TATP detectors particularly for security applications.

## 1. Introduction

Triacetone triperoxide (TATP) was discovered by Richard Wolffenstein more than 100 years ago. He already recognized its trimeric structure and emphasized the “tremendous explosibility” [1]. He also reported that the substance had destroyed his apparatus for elemental analysis. Due to its unpredictable behavior, it was never used commercially. Unfortunately, it became popular as a weapon of terrorists, because it is easy and cheap to manufacture based on starting materials, which are readily available as household chemicals. The structure of TATP is remarkable for an explosive, because it does not contain any nitro groups and shows only very weak UV absorbance. In addition, the density of 1.22 g cm^−3^ is completely uncharacteristic and TATP does not need any (metal-containing) igniter.

The detection of TATP can be accomplished by GC-MS [2,3], ion mobility spectrometry (IMS) [3,4,5], and indirectly, by detection of hydrogen peroxide after acidic cleavage [6,7,8,9,10] or UV irradiation [11,12]. In addition, TATP was analyzed by APCI-MS [13], ESI-MS [14], DESI-MS [15,16], IRMS [17], by HPLC-FT-IR [18], IR and Raman spectrometry [19]. Alternative photonic sensor devices for the detection of explosives have been discussed in [20], such as cavity ring down spectroscopy and quartz-enhanced photoacoustic spectroscopy (QEPAS). A general review was recently published by Burks and Hage [21]. However, most of the techniques either lack the mobility of the equipment, cost-effectiveness, speed, sufficient sensitivity or—which is particularly difficult in practice—the ultimate selectivity to avoid nerve-racking series of false positive detection events [20].

In contrast to TATP, for other explosives such as trinitrotoluene (TNT), several immunological methods have been presented in the past (e.g., [22,23,24,25]). Excellent sensitivity was obtained with immunoassays and in most cases the cross-reactivity problem was solved through careful hapten design [26,27,28,29]. 

A multitude of immunosensor platforms for the detection of explosives had been presented in the literature, e.g., [25,30,31,32,33,34,35,36,37]. A comprehensive review was published by Smith *et al*. in 2008 [38], including a large table of biosensors for explosives detection. Other reviews were compiled by Yinon [39], Singh [40], and Mitchell [41]. Only very few examples are shown for immunochemical gas-phase detection [42,43,44], which might indicate the significant challenge involved. Some systems have been designed to enable multianalyte detection based on microarray- or fiber-based immunosensors [45,46]. Novel recognition elements, such as molecularly imprinted polymers (MIPs) [47,48], aptamers [49], anticalins, nanobodies and other protein scaffolds have been developed [50]. Nevertheless, polyclonal and monoclonal antibodies are still by far the most popular and important binders for biosensor development. In the field of electronic noses, the application of biochemical recognition elements is still essentially nonexistent [51] or quite exotic [52]. In contrast, the application of antibodies for dipsticks or other rapid immunochemical tests is widespread. The application for the detection of TNT in water had been shown [53]. However, no immunochemical system for the detection of TATP is known, most likely due to lacking (immuno) reagents, which is a severe problem in the field [54]. Today, it becomes more and more obvious that highly selective recognition elements [50,55] are at least as important as the transducer in a biosensor system.

Recently, we tried to obtain monoclonal antibodies against triacetone triperoxide [56]. Unfortunately, the establishment of stable cell lines failed for unknown reasons. However, we could demonstrate that the production of mouse antibodies against TATP is possible. Now, we obtained polyclonal antibodies in rabbits, of which a full characterization and the application as competitive immunoassay are presented here.

## 2. Experimental Section

### 2.1. Reagents

Unless otherwise specified, chemicals and solvents were purchased from Sigma-Aldrich, Merck KGaA (Darmstadt, Germany), and J.T. Baker in the highest quality available. Acetone (picograde) and n-hexane (picograde) were supplied by LGC Standards. 3,3′,5,5′-Tetramethylbenzidine (TMB) (research grade) and Tween™20 (pure) were from Serva (Heidelberg, Germany). The buffers and solutions were prepared with ultrapure reagent water, which was obtained by running demineralized water (by ion exchange) through a Milli-Q^®^ ultrapure water purification system (Millipore Synthesis A10).

The proteins, used for hapten conjugations, were bovine serum albumin (BSA, for immunogen synthesis), fraction V, receptor grade, lyophilized, from Serva (11924, #080026) and horseradish peroxidase (HRP), EIA grade, from Roche (10814393001, #14265740). The anti-rabbit IgG was a polyclonal antibody to rabbit IgG [H&L] from goat, purified, purchased from Acris, Herford, Germany (R1364P, #19406).

The examined cross-reactants obtained from Sigma-Aldrich are listed with purity, order and lot number: ammonium nitrate, 99% (09890, #1376281); butanone, 99.5% (04380, #BCBB1352); 12-crown-4, 98% (194905, #MKBB0225G9); 18-crown-6, 99.5% (274984, #1311427); hydrogen peroxide, 30% (H1009, #S45604-507); nitroguanidine, containing about 25% water (N17351, #S31452); and 7-oxooctanoic acid, 98% (343625, #09017CE). Acetone, picograde, was purchased from LGC Standards (SO-1142-B040, #810903).

### 2.2. Safety Note

Only highly qualified personnel should work with TATP or other peroxide explosives and safety precautions must be strictly adhered to avoid hazardous situations. Furthermore, only small amounts of less than 100 mg should be synthesized and handled. TATP and other peroxides can detonate spontaneously, particularly under impact, friction, static electricity or temperature changes.

### 2.3. Synthesis of TATP, TATP Hapten, Immunogen and Enzyme Tracer

**Figure 1:**
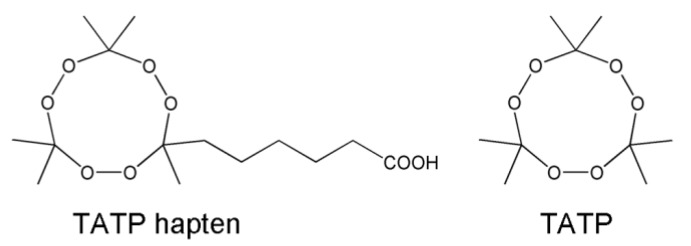
Chemical structures of the TATP hapten and TATP.

TATP and TATP hapten (Figure 1) were synthesized following the protocols described in Walter *et al*. [56].

The TATP immunogen was prepared by coupling the TATP hapten to bovine serum albumin (BSA). The method based on the *N*-hydroxysuccinimide chemistry with carbodiimide was performed similar to Tatake *et al*. [57]. Details are given in the supplementary material. Briefly, TATP hapten, *N*-hydroxysuccinimide (NHS), and *N,N′*-dicyclohexylcarbodiimide were mixed in anhydrous tetrahydrofuran in a molar ratio of 5:6:6. To ensure water-free conditions, first, a tip of a spatula of *N,N′*-disuccinimidyl carbonate [58] was added. After coupling the activated NHS ester of the TATP hapten to BSA in a sodium hydrogen carbonate buffer (130 mM, approx. pH 8) and purification of the TATP immunogen, a mean coupling ratio of 14 hapten molecules per BSA (molar ratio 38:1 in synthesis) was determined via MALDI-TOF-MS [59]—spectrum is provided as supplementary material Figure S-1—and the protein concentration of 7.8 g L^−1^ was photometrically determined.

In addition, a peroxidase conjugate consisting of TATP hapten (Figure 1) and horseradish peroxidase (HRP) was prepared. The procedure is similar to the synthesis of the immunogen. The molar ratio of TATP hapten, *N*-hydroxysuccinimide, and *N,N′*-dicyclohexylcarbodiimide was set to 1:2:2. MALDI-TOF-MS measurements showed a mean coupling ratio of about one hapten molecule per HRP (data not shown).

### 2.4. Immunization

The TATP-BSA conjugate was employed to immunize two, 9–12 week old (2–2.5 kg) rabbits with sub-cutaneous injections. The immunizations were performed at Eurogentec S.A. (Seraing, Belgium). The first immunization was done with 100 µg immunogen in 0.1 × PBS. The same amount was used for boosts 1–3, which were given on day 7, 10, and 18, respectively. Serum samples were taken on day 0 (pre-immune), 14 (boost 2), and 21 (boost 3). This follows the Eurogentec *Speedy 28-day protocol* with a proprietary adjuvant. With boost 4 on day 42 a customized schedule followed. After boost 5 on the 56th day, the animals were boosted every 28 days until the final boost 11 was administered on day 224. Boosts 4–11 were done with 50 µg TATP immunogen and with Incomplete Freund Adjuvant. Sera were collected 7 days after each injection and after boost 11 both rabbits were bled out. The sera were used to evaluate the titer and affinity maturation of the hapten-specific antibodies via ELISA.

### 2.5. Enzyme-Linked Immunosorbent Assay (ELISA)

A direct competitive TATP immunoassay (ELISA) was developed with sera of two rabbits. A brief description of the ELISA protocol is given here. Details are described in the supplement. Each well of the microtitration plate was coated with anti-rabbit IgG in PBS. The plates were shaken 18–24 h followed by the first washing step. Next, diluted rabbit serum (TATP antibody) was pipetted in the wells and incubated for 1 h. After another washing step, TATP standard solutions and dilutions of the HRP conjugate were added in triplicate and shaken for 30 min. The standards were prepared from a methanolic TATP stock solution by dilution in water. The plate was washed again, before a freshly prepared substrate (tetramethylbenzidine/hydrogen peroxide) solution was added to the wells. After incubation on a plate shaker and stopping the color reaction with sulfuric acid, the absorbance of each well was measured at 450 nm.

The relation between the absorbance and the analyte concentration in this competitive assay was calibrated using a four-parameter logistic function according to Dudley *et al*. [60] of mean values of the standards [61].

### 2.6. Syntheses of Cross-Reactants and Determination of Cross-Reactivities

The potential cross-reactivities (CR) of typical explosives or components of explosives as well as starting materials and structural analogues of TATP were determined: Acetone, ammonium nitrate, 12-crown-4, 18-crown-6, diacetone diperoxide (DADP), hexamethylene triperoxide diamine (HMTD), hexogen (RDX), hydrogen peroxide, nitroguanidine, nitropenta (pentrite, PETN), octogen (HMX), 7-oxooctanoic acid, TATP hapten, tri-butanone triperoxide, 2,4,6-trinitrotoluene (TNT), tri-2-pentanone triperoxide, and tri-3-pentanone triperoxide. DADP, HMTD, TATP hapten, and the cyclic triperoxides are not commercially available. Their synthesis is described in the supplement.

In most cases, stock solutions of the cross-reactants were gravimetrically prepared in concentrations of 1–10 g L^‑1^ in water, methanol, or dimethyl sulfoxide (DMSO), depending on their solubility. Subsequently, these solutions were diluted sequentially 1:10 in water to have seven aqueous solutions of each substance to obtain a calibration curve with pure water as the first calibrator and to test the cross-reactivity of the respective compounds in the competitive ELISA. A maximum of 1% organic solvent was accepted in the highest concentration of the dilution series. The ratio of the concentrations (mass and molar) of the potential cross-reactant and TATP at the point of inflection (parameter C or IC_50_) of the four-parameter logistic function (C_cross-reactant_ and C_TATP_) describes the cross-reactivity CR (Equation (1), in percent) [62].

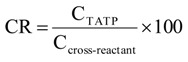
(1)


In case of absent or weak cross-reactivities, the parameter D of the ELISA curves of tested substances was fixed on the level of the TATP curve to facilitate the fitting of a sigmoidal curve. All cross-reactivities were examined with the serum of boost 7 of both rabbits (1:80,000). The HRP conjugate was used in a dilution of 1:100,000.

## 3. Results and Discussion

### 3.1. Antibody Development

Polyclonal TATP antibodies were developed in rabbits by injecting a TATP-BSA conjugate (immunogen) with a mean conjugation density of 14 haptens per protein. The immunization progress of both rabbits was monitored by ELISA, especially focused on IC_50_ and affinity development (data are shown in the supplementary material Table S-1).

The antibody titer was defined as the relative maximum absorbance (parameter A) of a TATP standard ELISA curve of each serum (data not shown). The first six boosts of both rabbits showed a small rise of the titer. After boost 6, the titer of rabbit 1 increased strongly and fell slightly after boost 8. The TATP antibody titer of rabbit 2 sera rose after boost 6 to boost 8 and decreased minimally after boost 9. It is evident that the course of the antibody titer is quite similar for the two rabbits. A short immunization protocol of up to 6 injections would not have been sufficient to obtain optimal antibody titers.

The IC_50_, which influences strongly the sensitivity of resulting TATP immunoassays, stagnated after boost 8 of both rabbit immunizations. Sera of rabbit 1 reached IC_50_ values below 0.3 µg L^−1^ and those of rabbit 2 close to 0.4 µg L^−1^ (supplementary material Table S-1). The affinities of the antibodies to TATP were calculated to ~1·× 10^9^ L mol^−1^ and ~0.7 × 10^9^ L mol^−1^ for rabbit 1 and rabbit 2, respectively (supplementary material Table S-1). The affinity was determined according to the lowest-IC_50_ method [29]. In brief, a series of competitive ELISA calibration curves were performed with decreasing concentrations of HRP conjugate *and* antibodies. The IC_50_ converges to a value, which allows the calculation of the affinity constant. The crucial point of this method is that it has to be performed two-dimensionally. The IC_50_ minimum can be finally confirmed, if neither a dilution of the antibody nor the dilution of the enzyme conjugate leads to a significant decrease of the IC_50_. The antibody affinity is the reciprocal IC_50_ after correction of the concentration of the TATP standard solution in the microtiter plate (due to dilution by adding HRP conjugate, assuming a fast equilibrium of the analyte) and conversion in molar dimensions. The error of the IC_50_ was used to estimate the error of the affinity constant. The results show that the immunization of both rabbits proceeded similarly and two almost identical polyclonal TATP sera were obtained. The Eurogentec *Speedy 28-day protocol*, which would have ended after boost 3, would have resulted in less sensitive antibodies and a much lower titer of antibodies. After boost 8, no further significant improvement was achieved.

### 3.2. Limit of Detection and Quantification Range

**Figure 2:**
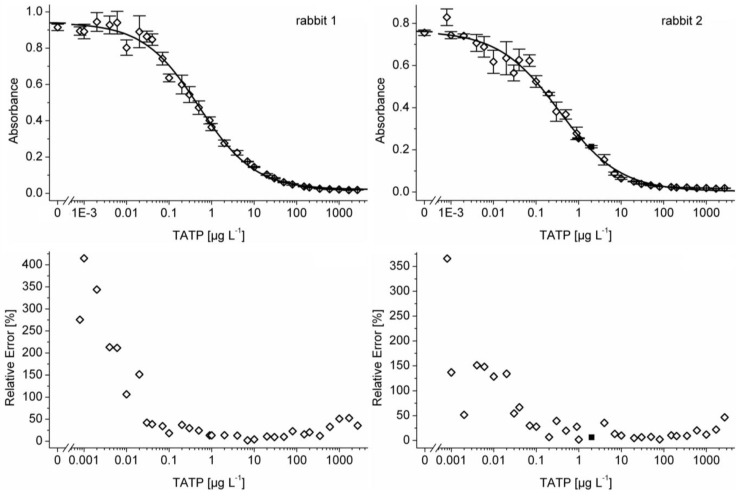
TATP calibration curves (four-parameter logistic function) and corresponding precision profiles obtained with 32 TATP standard solutions, including a blank of water. The LOD was 6 ng L^−1^ (rabbit 1) and 5 ng L^−1^ (rabbit 2). The IC_50_ of the sigmoidal curves obtained were 0.50 µg L^−1^ (rabbit 1) and 0.35 µg L^−1^ (rabbit 2). The slopes at the test midpoint (IC_50_) were 0.66 (rabbit 1) and 0.61 (rabbit 2). The sera (boost 11) were diluted 1:100,000; the HRP conjugate was diluted 1:300,000. Error bars in calibration curve: standard deviation (n = 3). Black square (rabbit 2: 2 µg L^−1^): One of three replicates was masked as outlier.

Figure 2 shows two ELISA calibration curves (32 standard solutions including water as blank) using dilutions of sera after boost 11 from both immunized rabbits. The limits of detection (LOD) were calculated subtracting three times the standard deviation (3 s definition) from the maximum absorbance (parameter A) of the corresponding curve. The mean detection limit for TATP of five separately determined ELISA curves was calculated to 9 ± 7 ng L^−1^ and 11 ± 5 ng L^−1^ for the sera of boost 11 of rabbit 1 and rabbit 2, respectively.

An important method to characterize the performance of an ELISA is to calculate a precision profile [63]. Therefore, an ELISA with 32 TATP standard dilutions was performed. The precision profile shows the quantification range of the assay on the basis of relative errors (Figure 2). Despite some problems with the standards at very low concentrations, the quantification over more than 4 orders of magnitude was possible, depending on the users’ acceptable maximum relative error. The quantification range based on the serum of rabbit 1 (boost 11) was between 0.03 µg L^−1^ and 500 µg L^‑1^, if a maximum relative error of 40% is acceptable. With the same limit, the assay based on the serum of rabbit 2 (boost 11) had a quantification range between 0.04 µg L^−1^ and 1,400 µg L^−1^, which is remarkable for a competitive immunoassay.

### 3.3. Cross-Reactivities

The selectivity of the TATP antibodies was evaluated by testing relevant compounds for cross-reactivity. First, some of the most common explosives like trinitrotoluene (TNT), hexogen (RDX), nitropenta (PETN), and octogen (HMX) were examined. Furthermore, the starting materials of the TATP synthesis and a few structurally related substances were studied. None of the explosives showed any cross-reactivity (<0.01%) in the ELISA, as shown in Table 1.

**Figure 3:**
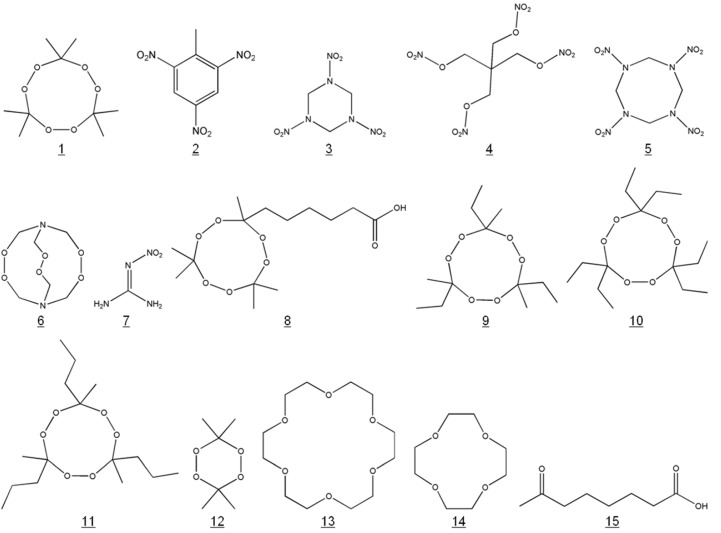
Chemical structures of potential cross-reactants (see Table 1 and Table 2; structures 9 and 11 represent examples of an isomer mixture).

Usually, metabolites of the analyte and its structural analogues are examined, too. In case of TATP, no metabolites or structurally similar compounds are known in the environment. Therefore, some other cyclic triperoxides were synthesized (Table 2 and Figure 3). Obviously, the TATP hapten (“derivatized” TATP) showed the strongest cross-reactivity: 340 ± 70% with rabbit 1 serum and 330 ± 10% with rabbit 2 serum. A minor cross-reactivity of the TATP antibodies was caused by tributanone triperoxide. Although only three (of six) methyl groups of TATP were formally exchanged to ethyl residues, the cross-reactivity was reduced to 2–4%. However, it has to be considered that the tested compound was a crude isomer mixture. Therefore, the resulting cross-reactivity might be caused by a single isomer. Each further extension of the carbon chains of TATP seems to reduce the cross-reactivity for these compounds rigorously, as demonstrated by tri-3-pentanone triperoxide and tri-2-pentanone triperoxide. It has to be noted that all these synthetic compounds do not have any practical and analytical relevance. All other tested substances in Table 2 revealed no significant cross-reactivity (<0.01%). Additionally, the starting materials of the TATP and TATP hapten synthesis were examined. Neither acetone or hydrogen peroxide, nor 7-oxooctanoic acid induced any cross-reaction.

**Table 1:** Cross-reactivities of the TATP antibodies for typical explosives or components of explosives.

			Rabbit 1	Rabbit 2	Rabbit 1	Rabbit 2
	Name	MW [g mol^−1^]	CR in %		molar CR in %	
1	TATP	222.24	100	100	100	100
2	TNT	227.13	<0.01	<0.01	<0.01	<0.01
3	RDX	222.12	<0.01	<0.01	<0.01	<0.01
4	PETN	316.14	<0.01	<0.01	<0.01	<0.01
5	HMX	296.16	<0.01	<0.01	<0.01	<0.01
6	HMTD	208.17	<0.01	<0.01	<0.01	<0.01
7	Nitroguanidine	104.07	<0.01	<0.01	<0.01	<0.01
	Ammonium nitrate (NH_4_NO_3_)	80.04	<0.01	<0.01	<0.01	<0.01

**Table 2:** Cross-reactivities of the TATP antibodies for starting materials and structural analogues of TATP (* mixed isomers).

			Rabbit 1	Rabbit 2	Rabbit 1	Rabbit 2
	Name	MW [g mol^−1^]	CR in %		molar CR in %	
8	TATP hapten	322.35	340 ± 70	330 ± 10	490 ± 100	470 ± 10
9	Tri-butanone triperoxide *	264.32	4	2	4	3
10	Tri-3-pentanone triperoxide	306.40	0.01	0.01	0.02	0.01
11	Tri-2-pentanone triperoxide *	306.40	<0.01	0.01	<0.01	0.01
12	Diacetone diperoxide (DADP)	148.16	<0.01	<0.01	<0.01	<0.01
13	18-Crown-6	264.32	<0.01	<0.01	<0.01	<0.01
14	12-Crown-4	176.21	<0.01	<0.01	<0.01	<0.01
15	7-Oxooctanoic acid	158.19	<0.01	<0.01	<0.01	<0.01
	Acetone (CH_3_COCH_3_)	58.08	<0.01	<0.01	<0.01	<0.01
	Hydrogen peroxide (H_2_O_2_)	34.01	<0.01	<0.01	<0.01	<0.01

## 4. Conclusions

TATP antibodies of extreme selectivity—no relevant cross-reactant could be found—and very high affinity (about 10^9^ L mol^−1^) could be obtained from long-term immunizations of rabbits. The hapten seems to have an appropriate structure to obtain useful antibodies against triacetone triperoxide (TATP). This could also be shown by long-term immunizations of mice [56]. Although both immunizations were performed with almost identical TATP-BSA conjugates (immunogens), the antibodies produced in rabbits had a more than thousand times higher affinity. The reason for this huge difference is still unclear. For a long time, researchers had speculated that rabbit (or rat) antibodies might be superior to those made in mice. However, to our knowledge, there are no documented cases for haptens in the literature (only for immunohistology: [64,65,66,67]). A relative simple rationale might be that mice have a less sophisticated immune system in comparison to rabbits. Our results suggest that for high-affinity antibodies against haptens, rabbits seem to be a superior species. We also could show that short-term immunizations, which are recommended by many custom antibody production services, may be significantly inferior to traditional long-term immunization protocols.

The determined limits of detection and the precision profile confirmed broad quantification range from the low nanogram per liter to around one milligram per liter in water. The absence of any relevant cross-reactants supports the extreme selectivity to TATP and proves successful hapten design. One of the major advantages of this ELISA is the direct detection of TATP based on its unique molecular structure without the detour via hydrogen peroxide, which is particularly susceptible to false positives.

The availability of highly sensitive and selective polyclonal TATP antibodies opens up new perspectives of TATP detection methods. Immunoassays or biosensor platforms, which had been presented for TNT and other explosives, e.g., based on surface-plasmon resonance, immunobeads, lab-on-a-chip systems, microarray biosensors, electrochemical setups and others might profit from the availability of high-quality immunoreagents for the selective detection of triacetone triperoxide. Considering the most recent developments in immunosensing [37], TATP might be detectable at sub-ppb levels in about 10 s.

## Supplementary Files

Supplementary File 1 PDF-Document (PDF, 225 KB)

## Supplementary Material

Supplementary material as noted in text. This material is available online: http://www.mdpi.com/2079-6374/1/3/93/.

## Material Availability

Samples of the antibodies can be obtained from the authors for research and testing purposes.
