# Supplementary material for: A Novel Immunoreagent for the Specific and Sensitive Detection of the Explosive Triacetone Triperoxide (TATP)
Source: Biosensors (Basel). 2011 Jul 7;1(3):93–106. doi: 10.3390/bios1030093 (PMC4264363; doi:10.3390/bios1030093)
Supplement: Supplementary File 1 — PDF-Document (PDF, 225 KB) [file biosensors-01-00093-s001.pdf]

Biosensors **2011**, *1*, S-1-S-6

OPEN ACCESS

biosensors

ISSN 2079-6374

www.mdpi.com/journal/biosensors/

Supplementary Material

A Novel Immunoreagent for the Specific and Sensitive Detection of the Explosive Triacetone Triperoxide (TATP)

Maria Astrid Walter [1], Ulrich Panne [1,2] and Michael G. Weller [1,*]

[1] BAM Federal Institute for Materials Research and Testing, Richard-Willstätter-Strasse 11, D-12489 Berlin, Germany; E-Mails: astrid.walter@bam.de (M.A.W.); ulrich.panne@bam.de (U.P.)

[2] Humboldt-Universität zu Berlin, Chemistry Department, Brook-Taylor-Strasse 2, D-12489 Berlin, Germany

* Author to whom correspondence should be addressed; E-Mail: michael.weller@bam.de; Tel.: +49-30-8104-1150; Fax: +49-30-8104-1157.

Summary: This supplement contains details for the synthesis of the TATP immunogen with a MALDI-TOF mass spectrum of this TATP-BSA conjugate and the synthesis of the peroxidase conjugate needed for ELISA (enzyme-linked immunosorbent assay). Also, a full description of the TATP ELISA protocol is comprised. Detailed information of the preparation of stock and standard solutions of the cross-reactants, including the syntheses of some compounds, is given. Moreover, the development of the IC_{50} and the affinity constants of both rabbits after each boost are shown.

Safety Note

Only highly qualified personnel should work with TATP or other peroxide explosives and safety precautions must be strictly adhered to avoid hazardous situations. Furthermore, only small amounts of less than 100 mg should be synthesized and handled. TATP and other peroxides can detonate spontaneously, particularly under impact, friction, static electricity or temperature changes.

Synthesis of Immunogen

The TATP immunogen was prepared by coupling the TATP hapten to bovine serum albumin (BSA) via *N*-hydroxysuccinimide (NHS) chemistry with dicyclohexylcarbodiimide (DCC) according to Tatake *et al.* [1]. For this purpose, *N*-hydroxysuccinimide (8.2 µmol, 0.97 mg) in anhydrous tetrahydrofuran (THF, 22 µL) was added to HPLC-purified and dried TATP hapten (6.8 µmol, 2.2 mg), which also was dissolved in anhydrous THF (50 µL). A small amount of *N*,*N'*-disuccinimidyl

carbonate (DSC, tip of a spatula) was given to the stirring mixture to ensure a water-free solution [2]. After addition of *N,N′*-dicyclohexylcarbodiimide (DCC, 8.2 µmol, 1.71 mg in 22 µL anhydrous THF) the reaction batch stirred over night at room temperature and protected from light. This procedure was performed under argon atmosphere and the molar ratio of hapten, NHS and DCC was 5:6:6. In order to separate the solution with the activated NHS ester of the TATP hapten from the precipitate (mainly dicyclohexyl urea), the mixture was centrifuged at 15 °C and 16,400 rpm for 10 min. The supernatant was added dropwise to a sodium hydrogen carbonate buffered BSA solution (0.18 µmol, 12 mg in 899 µL 130 mM $NaHCO_3$, pH 8) and stirred for 3.5 h at room temperature. The purification of the TATP immunogen was performed on a PD-10 desalting column (with Sephadex™ G-25, GE Healthcare) using diluted phosphate buffered saline (PBS, 1 mM sodium dihydrogen phosphate ($NaH_2PO_4 \cdot 2\ H_2O$), 7 mM sodium hydrogen phosphate ($Na_2HPO_4 \cdot 2H_2O$), 14.5 mM sodium chloride (NaCl), pH 7.6) as the eluent. The conjugate was collected manually in a UV-transparent 96-well microtitration plate (UV-Star®-96 Microplate, 96 Well, Greiner Bio-One, Frickenhausen, Germany) and measured at 280 nm in a MTP reader. A mean coupling ratio of 14 hapten molecules per BSA (molar ratio 38:1 in synthesis) was determined via MALDI-TOF-MS [3] (Figure S-1) and a protein concentration of 7.8 g L^{-1} was photometrically determined at 280 nm based on a BSA calibration.

Figure S-1. MALDI-TOF-MS spectra (detail) of non-conjugated BSA and TATP-BSA conjugate. The spectra were normalized to a baseline by subtracting the average of the signal in the range of m/z 53,000–63,000 from the measured values and smoothed by adjacent averaging of 300 points (TATP hapten, MW: 322.25 g mol^{-1}, coupling ratio hapten:BSA = 14:1).

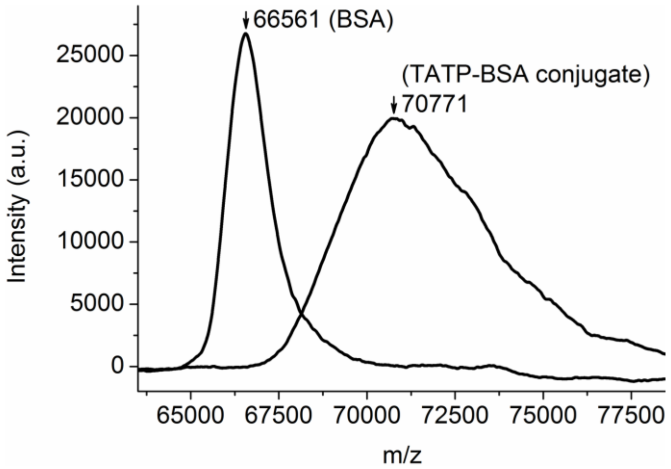

Synthesis of HRP Conjugate

To perform an ELISA (enzyme-linked immunosorbent assay), an enzyme conjugate consisting of TATP hapten and horseradish peroxidase (HRP) is needed. The protocol is similar to the synthesis of the immunogen (see above). The hapten-NHS ester was prepared in anhydrous *N,N′*-dimethylformamide (DMF) without adding DSC. The molar ratio of TATP hapten (4.3 µmol, 1.37 mg in 80 µL DMF), NHS (8.5 µmol, 1.01 mg in 31.5 µL DMF), and DCC (8.5 µmol, 1.77 mg in 28.3 µL DMF) was set to 1:2:2. 62 µL of the centrifuged reaction mixture was dripped into a sodium bicarbonate buffered HRP solution (0.089 µmol, 3.9 mg in 630 µL 130 mM $NaHCO_3$, approx.

pH 8), which corresponds to a ratio of 21:1 of TATP hapten to protein in the reaction mixture. MALDI-TOF-MS [3] measurements showed a mean coupling ratio below 1 hapten molecule per HRP. The concentration of the purified HRP conjugate was determined to be 4.2 g L^{-1}, which finally was diluted 1:2 with Peroxidase Conjugate Stabilizer (Guardian™, Thermo Scientific) and stored at 4 °C.

Enzyme-Linked Immunosorbent Assay (ELISA)

ELISA was performed at room temperature in transparent microtiter plates with a high binding surface (Thermo Scientific/Nunc Immuno 96 MicroWell™ Solid Plates MaxiSorp™, C96, No. 446612). Each well was coated with 200 µL of 0.9 mg L^{-1} anti-rabbit IgG in PBS (10 mM sodium dihydrogen phosphate ($NaH_2PO_4 \cdot 2H_2O$), 70 mM sodium hydrogen phosphate ($Na_2HPO_4 \cdot 2H_2O$), 145 mM sodium chloride (NaCl), pH 7.6). The Parafilm®-covered plates were shaken 18–24 h at 750 rpm followed by the first washing step. A 96-channel plate washer (ELx405 Select™, BioTek Instruments, Bad Friedrichshall, Germany) was used to wash the plates three times with washing buffer (washing buffer: 0.75 mM potassium dihydrogen phosphate (KH_2PO_4), 6.25 mM dipotassium hydrogen phosphate (K_2HPO_4), 0.025 mM sorbic acid potassium salt, 0.05% (v/v) Tween™20, pH 7.6). Next, 200 µL rabbit sera diluted in PBS containing 0.005% (w/v) BSA were pipetted in the wells and was incubated by shaking for 1 h. The required dilutions depended on the stage of the immunization and the type of experiment, varying from 1:5,000 to 1:600,000. Typically, serum of boost 7 was diluted 1:80,000 and serum after boost 11 was diluted 1:100,000. Thereafter, another washing step was performed and 200 µL of TATP standard solution was added in triplicate. Finally, 50 µL of diluted HRP conjugate was added. The standards (seven or 31, depending on the experiment) were prepared from a methanolic TATP stock solution of 10 g L^{-1}, by dilution in water, starting from 500 µg L^{-1} to 0.001 µg L^{-1} (seven calibrators) 2,700 µg L^{-1} to 0.0008 µg L^{-1} (31 calibrators). A blank of water was used as the first point in the calibration curve. The stabilized HRP conjugate (2.1 g L^{-1}) was diluted from 1:20,000 to 1:600,000, depending on the experiment and the immunization stage. For boost 7, the HRP conjugate was diluted 1:100,000 and for boost 11 the dilution was 1:300,000, for example. After shaking standards and HRP conjugate for 30 min, the plate was washed again. Directly afterwards, 200 µL substrate solution was added to the wells and incubated on a plate shaker. The substrate solution for one plate was always freshly prepared with 21.6 mL citrate buffer (220 mM potassium dihydrogen citrate, 0.5 mM sorbic acid potassium salt, pH 4), 8.34 µL H_2O_2 (30%) and 540 µl TMB solution (according to Frey *et al.* [4]: 40 mM 3,3′,5,5′-tetramethylbenzidine (TMB) and 8 mM tetrabutylammonium borohydride in *N*,*N*′-dimethylacetamide). The immobilized HRP conjugate and its substrates TMB and hydrogen peroxide cause the formation of a blue color, which turns to yellow, when 100 µL sulfuric acid (1 M H_2SO_4) is added to stop the reaction after 10–45 min, depending on the ELISA conditions. A microplate spectrophotometer (SpectraMax Plus^{384}, Molecular Devices) controlled by Softmax® Pro 5.3 software measured the absorbance of each well at 450 nm using 620 nm as reference.

Synthesis of Cross-reactants

The synthesis of diacetone diperoxide (DADP), hexamethylene triperoxide diamine (HMTD), and other cyclic triperoxides was needed to determine their potential cross-reactivities (CR).

DADP (CAS No. 1073-91-2) was synthesized following the procedure of Dubnikova *et al*. [5]. Washed and dried TATP (0.47 mmol) was dissolved in anhydrous chloroform (500 µL) with a catalytic amount of *p*-toluenesulfonic acid (~0.002 mmol, <5 g L^{-1}). The mixture was incubated for two weeks at room temperature and protected from light. Subsequently, the solvent was evaporated and the product was recrystallized from hot methanol. After the second recrystallization, the DADP crystals were kept in the methanolic mother liquor to avoid potential decomposition. A 10 g L^{-1} DADP stock solution in methanol was gravimetrically prepared without completely removing methanol from the crystals (Therefore, the concentration is only an estimated value.). The success of the DADP formation was proven via X-ray crystallography and NMR (data not shown).

HMTD (CAS No. 283-66-9) was synthesized according to Wierzbicki and Cioffi [6]. Hexamethylenetetramine (0.75 mmol) was dissolved in aqueous hydrogen peroxide (9.24 mmol, 30%). The mixture was cooled to 0 °C and after cautiously adding solid citric acid (0.92 mmol), the temperature was slowly increased to room temperature. Five days later, the formed HMTD crystals were thoroughly washed with water and ice-cold methanol and then dried carefully under reduced pressure. X-ray crystallography (data not shown) verified the resulting product in comparison to Schaefer *et al*. [7]. A 1 g L^{-1} stock solution was prepared gravimetrically in dimethyl sulfoxide (DMSO), because of the poor solubility of HMTD in numerous solvents.

Analogous to the production of TATP, three other cyclic triperoxides (raw isomer mixtures) were synthesized from butanone, 2-pentanone and 3-pentanone instead of acetone. As described before, 3 mmol of the corresponding ketone was mixed with hydrogen peroxide (1.5 mmol, 30%) and sulfuric acid (0.015 mmol, 2 M) at 0 °C. After 48 h, the stock solutions were prepared by dissolving the reaction mixtures completely in 10 ml methanol each. A TATP synthesis was carried out in parallel. Its yield of about 60% was determined with ELISA in comparison with the purified TATP. The concentrations of the other three triperoxides (tri-butanone triperoxide, tri-2-pentanone triperoxide, and tri-3-pentanone triperoxide) were assumed to be similar to the TATP synthesis. Because of the lack of crystals and difficulties to purify the desired triperoxide products, X-ray studies were unfeasible, but NMR measurements suggested the successful synthesis of the respective products (data not shown).

Stock solutions of 10 g L^{-1} acetone (picograde, CAS No. 67-64-1, SO-1142-B040 LGC Standards, #810903), hydrogen peroxide (30%, CAS No. 7722-84-1, H1009 Sigma-Aldrich, #S45604-507), 7-oxooctanoic acid (98%, CAS No. 14112-98-2, 343625 Sigma-Aldrich, #09017CE), 12-crown-4 (98%, CAS No. 294-93-9, 194905 Sigma-Aldrich, MKBB0225G9), and ammonium nitrate (99%, CAS No. 6484-52-2, 09890 Sigma-Aldrich, #1376281) and 1 g L^{-1} 18-crown-6 (99.5%, CAS No. 17455-13-9, 274984 Sigma-Aldrich, #1311427) in water were prepared gravimetrically. Nitroguanidine was weighed out, taking into account that it contains about 25% water (CAS No. 556-88-7, N17351 Sigma-Aldrich, #S31452), and a 10 g L^{-1} stock solution in DMSO was obtained. Technical trinitrotoluene (TNT, CAS No. 118-96-7), hexogen (RDX, CAS No. 121-82-4), nitropenta (pentrite, PETN, CAS No. 78-11-5), and octogen (HMX, CAS No. 2691-41-0) were dissolved in methanol/DMSO (1:1, v/v) obtaining a 5 g L^{-1} stock solution. A TATP hapten stock solution in

methanol, estimated to be ~1 g L^{-1}, was diluted 1:100 in water to approximately 10 mg L^{-1}. This solution of TATP hapten was compared with a 10 mg L^{-1} TATP solution by HPLC (conditions specified in Walter *et al*. [8]) and thus, the concentration was calculated to be 8.88 mg L^{-1}. A series of six consecutive 1:10 dilutions in water was prepared to determine the cross-reactivity of the TATP hapten. Furthermore, all other stock solutions were also diluted sequentially 1:10 in water to have seven aqueous solutions of each substance. The dilution series of acetone, hydrogen peroxide, and 7-oxooctanoic acid started with 10 g L^{-1} as the highest concentration; DADP, 18-crown-6, 12-crown-4, TNT, RDX, HMTD, ammonium nitrate, and nitroguanidine with 100 mg L^{-1}, and the dilution series of PETN and HMX started with 50 mg L^{-1} and 10 mg L^{-1}, respectively. The three other cyclic triperoxides and the TATP control synthesis were diluted 1:200 and 1:2,000, respectively, independent of their concentration.

Details on Antibody Development

Table S-1. Development of the IC_{50} of the serum of both rabbits after each boost (optimized ELISA) and calculated antibody affinity (lowest-IC_{50} method). Error: standard deviation (n = 3).

	**Boost no.**	**Dilution serum**	**HRP conjugate**	**IC_{50} [µg L^{-1}]**	**Affinity [L mol^{-1}]**
**Rabbit 1**					
	2	1:5,000	1:20,000	140 ± 45	0.002 ± 0.001 × 10^9
	3	1:20,000	1:40,000	37.8 ± 13.7	0.007 ± 0.003 × 10^9
	4	1:120,000	1:30,000	3.59 ± 1.36	0.077 ± 0.029 × 10^9
	5	1:120,000	1:30,000	1.50 ± 0.21	0.185 ± 0.026 × 10^9
	6	1:40,000	1:180,000	0.859 ± 0.170	0.323 ± 0.064 × 10^9
	7	1:200,000	1:100,000	0.608 ± 0.364	0.457 ± 0.273 × 10^9
	8	1:500,000	1:600,000	0.225 ± 0.026	1.234 ± 0.143 × 10^9
	9	1:300,000	1:600,000	0.297 ± 0.064	0.934 ± 0.200 × 10^9
	10	1:300,000	1:600,000	0.274 ± 0.108	1.015 ± 0.402 × 10^9
	11	1:300,000	1:600,000	0.268 ± 0.074	1.037 ± 0.286 × 10^9
**Rabbit 2**					
	2	1:15,000	1:40,000	78.6 ± 21.2	0.004 ± 0.001 × 10^9
	3	1:40,000	1:30,000	16.8 ± 2.7	0.017 ± 0.003 × 10^9
	4	1:120,000	1:30,000	1.91 ± 0.63	0.146 ± 0.048 × 10^9
	5	1:120,000	1:30,000	1.25 ± 0.09	0.222 ± 0.015 × 10^9
	6	1:120,000	1:30,000	0.850 ± 0.130	0.327 ± 0.050 × 10^9
	7	1:100,000	1:300,000	0.535 ± 0.167	0.519 ± 0.162 × 10^9
	8	1:200,000	1:600,000	0.400 ± 0.061	0.694 ± 0.105 × 10^9
	9	1:600,000	1:600,000	0.426 ± 0.050	0.653 ± 0.076 × 10^9
	10	1:600,000	1:600,000	0.447 ± 0.084	0.621 ± 0.116 × 10^9
	11	1:300,000	1:600,000	0.407 ± 0.072	0.683 ± 0.121 × 10^9

References

1. Tatake, J.G.; Knapp, M.M.; Ressler, C. Synthesis and characterization of protein and polylysine conjugates of sulfamethoxazole and sulfanilic acid for investigation of sulfonamide drug allergy. *Bioconjug. Chem.* **1991**, *2*, 124-132.
2. Weller, M.G.; Weil, L.; Niessner, R. Determination of triazine herbicides by ELISA—Optimization of enzyme tracer synthesis. *Fresenius J. Anal. Chem.* **1992**, *343*, 51-52.
3. Bahlmann, A.; Weller, M.G.; Panne, U.; Schneider, R.J. Monitoring carbamazepine in surface and wastewaters by an immunoassay based on a monoclonal antibody. *Anal. Bioanal. Chem.* **2009**, *395*, 1809-1820.
4. Frey, A.; Meckelein, B.; Externest, D.; Schmidt, M.A. A stable and highly sensitive 3,3′,5,5′-tetramethylbenzidine-based substrate reagent for enzyme-linked immunosorbent assays. *J. Immunol. Meth.* **2000**, *233*, 47-56.
5. Dubnikova, F.; Kosloff, R.; Almog, J.; Zeiri, Y.; Boese, R.; Itzhaky, H.; Alt, A.; Keinan, E. Decomposition of triacetone triperoxide is an entropic explosion. *J. Am. Chem. Soc.* **2005**, *127*, 1146-1159.
6. Wierzbicki, A.; Cioffi, E. Density functional theory studies of hexamethylene triperoxide diamine. *J. Phys. Chem. A* **1999**, *103*, 8890-8894.
7. Schaefer, W.P.; Fourkas, J.T.; Tiemann, B.G. Structure of hexamethylene triperoxide diamine. *J. Am. Chem. Soc.* **1985**, *107*, 2461-2463.
8. Walter, M.A.; Pfeifer, D.; Kraus, W.; Emmerling, F.; Schneider, R.J.; Panne, U.; Weller, M.G. Triacetone triperoxide (TATP): Hapten design and development of antibodies. *Langmuir* **2010**, *26*, 15418-15423.

© 2011 by the authors; licensee MDPI, Basel, Switzerland. This article is an open access article distributed under the terms and conditions of the Creative Commons Attribution license (http://creativecommons.org/licenses/by/3.0/).
